# PGMD: a comprehensive manually curated pharmacogenomic database

**DOI:** 10.1038/tpj.2015.32

**Published:** 2015-05-05

**Authors:** A Kaplun, J D Hogan, F Schacherer, A P Peter, S Krishna, B R Braun, R Nambudiry, M G Nitu, R Mallelwar, A Albayrak

**Affiliations:** 1 BIOBASE GmbH, Halchtersche Strasse 33, Wolfenbüttel, Germany

## Abstract

The PharmacoGenomic Mutation Database (PGMD) is a comprehensive manually curated pharmacogenomics database. Two major sources of PGMD data are peer-reviewed literature and Food and Drug Administration (FDA) and European Medicines Agency (EMA) drug labels. PGMD curators capture information on exact genomic location and sequence changes, on resulting phenotype, drugs administered, patient population, study design, disease context, statistical significance and other properties of reported pharmacogenomic variants. Variants are annotated into functional categories on the basis of their influence on pharmacokinetics, pharmacodynamics, efficacy or clinical outcome. The current release of PGMD includes over 117 000 unique pharmacogenomic observations, covering all 24 disease superclasses and nearly 1400 drugs. Over 2800 genes have associated pharmacogenomic variants, including genes in proximity to intergenic variants. PGMD is optimized for use in annotating next-generation sequencing data by providing genomic coordinates for all covered variants, including Single Nucleotide Polymorphisms (SNPs), insertions, deletions, haplotypes, diplotypes, Variable Number Tandem Repeats (VNTR), copy number variations and structural variations.

## Introduction

As Next Generation Sequencing (NGS) becomes more affordable, effective and comprehensive, personalized medicine gets closer to practical implementation. However, routine clinical use of NGS data still faces a number of challenges in regulation, integration into clinical information systems, data management, and in the interpretation of sequencing results to identify actionable genetic variants. There are two major categories of such variants that often overlap: variants responsible for the patient's condition and variants affecting drug response. Genetic aberrations belonging to the first category of causal disease variants have been the main focus of research and clinical communities. As a result, there are multiple resources available for evaluating the likelihood that a given lesion is either harmless or pathogenic. Although they differ by scope, deposition and access policies, notable resources of curated disease variants include the following: the manually curated Human Gene Mutation Database,^1^ which currently represents the most comprehensive source of information on germline disease-causing mutations and disease-associated polymorphisms; Online Mendelian Inheritance in Man (OMIM) (similar in scope to Human Gene Mutation Database but including significantly fewer variants,^2^); ClinVar (a relatively new public archive of reports that lists relationships between human variations and phenotypes with supporting evidence^3^); Catalogue of somatic mutations in cancer (COSMIC);^4^ and multiple locus-specific databases.

The second, potentially more actionable category of genetic variants, those affecting drug response and which are therefore directly applicable for determining personalized treatment strategy, is not covered as well by currently available data resources. Two resources, the Drug Gene Interaction Database^5^ and the Comparative Toxicogenomics Database,^6^ aggregate information about relationships between genes, diseases and drugs or chemicals but neither considers how specific genetic alteration may affect response to a drug or chemical. The Pharmacogenomics Knowledge Base (ref. 7), a manually curated resource containing information on pharmacogenomic variants in hundreds of genes and related drugs, summaries about important pharmacogenes, and pharmacogenomic pathways does consider the effect of specific genetic alterations. However, although its scope and breadth of information is widely recognized in the pharmacogenomics community, it cannot be easily cross-referenced to NGS data because of a lack of genomic coordinates for many of the described variants. Multiple additional factors, including the incomplete presentation of complex genotypes and star alleles, a lack of linkage disequilibrium information and emphasis on established pharmacogenes, leave space for independent curation and data presentation efforts. Here we present the PharmacoGenomic Mutation Database (PGMD), a manually curated database of drug response variants. The aim of this database is to provide a comprehensive resource for all variants that have been reported to have a pharmacogenomic effect in human studies and to describe those variants by exact genomic location and sequence alterations for application to NGS data analysis. The database is designed to contain extensive information as evidence for these associations, including information on resulting phenotype, drugs administered, patient population, study design, disease context, statistical significance and provenance of this information. Online access to PGMD is free for registered users from academic institutions. Access for commercial users and a variety of download options is available via paid subscription.

## Materials and methods

### Content acquisition

The primary source of PGMD content is the peer-reviewed scientific literature. Relevant articles are identified by a combination of manual selection and automated querying of PubMed. The current release (2014.4) contains 5904 references. A secondary source of content stems from the pharmacogenomic associations that have been reported to the FDA and EMA by drug manufacturers. Relevant content is extracted from FDA and EMA drug labels.^8^

PGMD data are manually curated by a team of scientific curators. To ensure high fidelity between original publication content and what is reported in PGMD, data are entered via a semi-dual curation process. Core data values such as genotype, statistical significance and specific drug response (phenotype) are entered independently by two separate curation scientists before being compared and compiled by a scientific editor. Further details, including patient ethnicity, age, drug(s) administered and disease are captured by one of the pair of curators and subsequently reviewed by the same scientific editor. To ensure standardization across records, most categories of data, including phenotype, disease Medical Subject Headings (MeSH), drugs (DrugBank, PubChem, MeSH) and many supporting details, are captured using controlled vocabularies.

PGMD curators manually determine Human Genome Variation Society (HGVS) nomenclature to be associated with each genetic variation, or gather this information from National Center for Biotechnology Information (NCBI)'s Short Genetic Variations database (dbSNP).^9^ Information crucial to cross-referencing pharmacogenetic annotations to NGS data is often only partially found in the literature and must be resolved manually by mapping to the human reference assembly via NCBI. In studies where information crucial to identifying genomic location is missing, the policy is to personally communicate with the authors to obtain the necessary details to facilitate mapping to genomic coordinates.

## Results

### Content scope

The basic unit of PGMD is the variant or haplotype, which is represented in the online delivery model as a Variant Report or Haplotype Report. Overview information is provided for each variant, when possible, including the variant type and class, reference allele, allele frequency and more. Overview information is followed by the list of pharmacogenomic studies curated for the variant or haplotype. Each study breaks down into a set of observations, with each observation including five core fields of data: a genotype, haplotype, diplotype and so on for more complex variants; a phenotype; the administered drug; statistical significance of the association; and the source of the data. Additional data fields are captured when available, including treatment and sample source details, disease state, population details of the patients, total study size and more. A complete list of data fields is provided in Supplementary Table 1. When available, HapMap^10^ linkage disequilibrium data are provided as population-based D′ and r^2^ scores providing insight into potentially linked causal variants.

PGMD data trends, which become visible while querying the complete database, highlight the impartial nature of the content acquisition process and the broad scope of the data. Variants found in PGMD are annotated into several functional categories. A total of 13 454 variants have a pharmacodynamic role, where variation at the given site or haplotype has led to altered impact of a drug on the patient, including adverse events; 1950 variants have a pharmacokinetic role, where variation at the given site or haplotype has led to differential absorption, distribution, metabolism and excretion of the drug; 2865 variants alter observed clinical outcomes of treatments. Assessment of clinical outcome is complex and could include four types of measures: patient-reported outcome, clinician-reported outcome, observer-reported outcome and performance outcome according to FDA classification. A small subset of data found within PGMD (671 variants) falls into the “molecular assay” category, where a parameter could only be measured *in vitro*, and therefore is the only group of PGMD variants that is not based on human *in vivo* studies.

Lack of bias or preference in reference screening for disease indication of a drug, specific disease within a study, or the presumed role of the associated gene in a pathological process or drug metabolism has led to a wide scope of coverage by PGMD. Within the database, a total of 480 diseases are covered, representing all 24 MeSH^11^ disease superclasses (Table 1) recognized by the American Medical Association. A total of 1390 drugs have been captured for the various specified disease indications, including drugs in the process of FDA approval, stages 2–4. PGMD's drug report provides comprehensive information about each drug, including metabolizing enzymes of each drug, known targets of each drug, and related clinical trials.

Not restricted to variants in key Absorption, Distribution, Metabolism and Excretion genes,^12^ which receive heavy attention from the scientific community and are well covered by targeted gene sequencing panels provided by major vendors, PGMD also captures variants from pharmacogenomic studies in other genes and intergenic regions, providing coverage to less studied regions of the genome for research of less established pharmacogenomic markers.

Given the unbiased coverage of highly studied and understudied pharmacogenetic associations, PGMD has coverage of a wide variety of genes. A total of 2802 genes contain reported pharmacogenomic variants; among them 689 encode drug targets and 121 belong to drug-metabolizing pathways. This excludes many variants that fall completely outside of genic regions, which are distinctively associated with the genes that surround such variants. The current release of PGMD contains 3796 genes that are classified as nearby genes for intergenic variants. By providing surrounding genes for intergenic variants, PGMD facilitates exploration of hypotheses relating to potential gene regulation roles of a variant.

### Delivery

PGMD is available via an online interface, and as a download via either a MySQL database or as a set of flat files. PGMD is also incorporated into Genome Trax, a genomic annotation and analysis database.^13^

**Figure 1:**
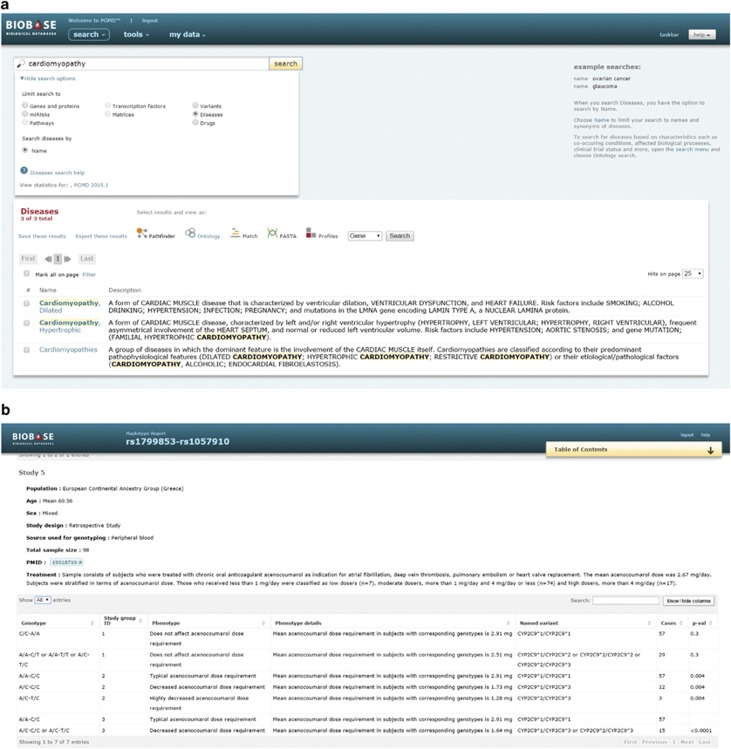
PharmacoGenomic Mutation Database (PGMD) online interface. (**a**) Pharmacogenomic variants can be retrieved by focus diseases, drugs, genes or particular variants. (**b**) Screenshot of a part of variant report showing one of the annotations associated with haplotype rs1799853–rs1057910.

The PGMD interface (Figure 1) allows searching of pharmacogenomic variants individually or in bulk by genomic coordinates, identifiers or amino acid changes. Additional searchable and uploadable categories include genes, proteins and miRNAs containing the variants, as well as affected diseases and differentially responding drugs. Examples of search for different terms and instructions for downloading search results can be found in Supplementary Tutorial. PGMD's online interface has been integrated with the interface of PROTEOME and TRANSFAC databases,^14, 15^ allowing for an intuitive transition for experienced users, and cross-referencing to millions of entries of these resources, including reports related to genes, diseases, drugs, pathways, variants and more. Searches by the aforementioned entities across individual databases or their combinations generate reports with links providing access from one entity's report to the next; however, access to PROTEOME and TRANSFAC content requires a subscription to these databases.

**Figure 2:**
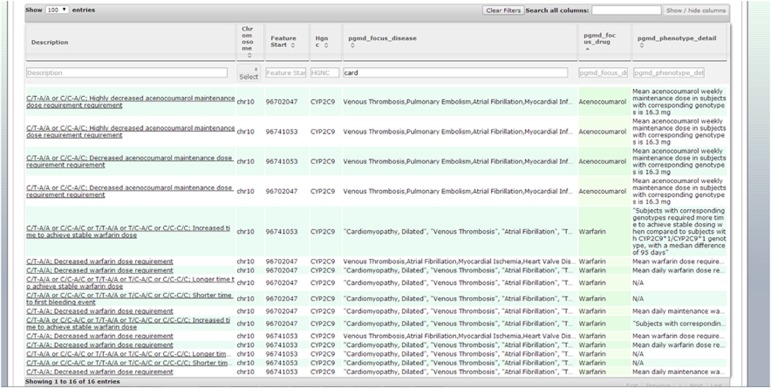
Pharmacogenomic annotation of Next Generation Sequencing data by Genome Trax using PGMD annotation track. A subset of 61 available data fields (Supplementary Table 1) are shown for each of the annotated variants, additional fields may be added to the view via *Show/hide columns*.

In addition to PGMD data, Genome Trax includes data from multiple additional annotation tracks, including Human Gene Mutation Database, ClinVar, COSMIC, TRANSFAC, PROTEOME and more. The addition of PGMD allows users to intuitively add pharmacogenomic variants to a genome of interest and perform filtering of the variants that match their subject on the basis of disease, drugs administered, ethnicity, statistical significance and more (Figure 2, Supplementary Table 1).

Many academic, clinical or commercial institutions have developed their own NGS data analysis pipelines, created for alignment, variant calling, quality control of calls, annotation of public and private data sets, and other advanced functions such as cohort and trio analyses. A downloadable database is required for the integration of pharmacogenomic data into such analysis pipelines. PGMD offers two such options; one is a MySQL database that includes all pharmacogenomic variants available through the online option, as well as supplementary data such as reference alleles from Genome Reference Consortium Human Builds, allele frequencies from sources such as HapMap, the 1000 Genomes Project^16^ and the Exome Sequencing Project,^17^ and data on linkage disequilibrium correlating with pharmacogenomic variants. Users also have the option of a flat file in the form of Tab-separated values, in which simple variants such as SNPs and Indels have been separated into one file with all relevant data columns, and more complex variants such as haplotypes, repeats and structural variations have been included in a second file (Supplementary Table 2).

## Discussion

The PGMD is a unique resource that has aggregated the literature on drug response in patients into one easily accessible knowledgebase. By allowing a user to quickly overlay the previously observed correlations, we have made it possible to provide meaning to a patient's genome in a clinical context, helping guide both clinical trials and potential treatment of possibly harmful drugs on an individual basis. The online user interface enables the database to be easily searched by drug, disease, gene, haplotype or variation and also provides information on SNPs that are in linkage disequilibrium with reported pharmacogenomic variants. To make the database useful for exome or whole-genome screening, we have developed algorithms that allow matching of the entries in the database against a sampled subject's variants, taking into account the fact that, in many cases, haplotypes need to be matched, exact nucleotide changes must be considered and complex star alleles must be resolved properly.

We plan to continue our efforts of further development of PGMD in several directions. For example, the current scope of PGMD does not cover reports of nonsignificant variants—that is, variants reported in the peer-reviewed literature to not have significant pharmacogenomic effects. We plan to extend our curation to include such reports, especially in cases of controversial clinical evidence, where they do contradict a ‘significant finding' included in PGMD.

PGMD's web-based interface allows for querying for variants based off of the gene it is contained within, the drugs that were administered to the patients in the study, and the disease that the patients in the study had. Therefore, a disease search does not identify all studied variants pertaining to all drugs that treat (or may treat) a given indication. Expanding PGMD ontology search to incorporate drug–disease relationships, and search accordingly, is another future goal.

At this time, no meta-analysis has been conducted on variants found within PGMD. Such a feature would allow a user to make an assessment on the best treatment regimen for a patient, given (possibly conflicting) associations found for a drug, via a weighting algorithm that factors in sample sizes, statistical significance of each observation, patient vs study population details, etc. The described “on the fly” analysis algorithm would be particularly beneficial for clinical reporting. Although we do not have immediate plans of development of meta-analysis tools for PGMD or Genome Trax web interface, future integration with Ingenuity Variant Analysis, as well as with the upcoming Clinical Decision Support application, will cover this gap. Potential applications of the integration include annotation of variants, annotation of haplotypes and aggregation of multiple, possibly conflicting findings into a decisive conclusion on best possible treatment.

## Figures and Tables

**Table 1:** Number of pharmacogenomic associations and variants, by MeSH disease class

*MeSH disease class*	*Total unique observations*	*Total unique variants*
Bacterial infections and mycoses	5846	754
Behavior and behavior mechanisms	109	35
Cardiovascular diseases	9010	1808
Congenital, hereditary and neonatal diseases and abnormalities	901	243
Digestive system diseases	14 476	1139
Endocrine system diseases	3593	692
Eye diseases	581	75
Female urogenital diseases and pregnancy complications	7091	986
Hemic and lymphatic diseases	3915	947
Immune system diseases	18 409	5380
Male urogenital diseases	6639	864
Mental disorders	14 919	2369
Musculoskeletal diseases	7297	3327
Neoplasms	31 941	4999
Nervous system diseases	5410	1650
Nutritional and metabolic diseases	3656	499
Otorhinolaryngologic diseases	322	44
Parasitic diseases	31	14
Pathological conditions, signs and symptoms	2069	399
Respiratory tract diseases	9397	1511
Skin and connective tissue diseases	13 036	4526
Stomatognathic diseases	320	46
Substance-related disorders	799	143
Virus diseases	9145	896
Total unique^a^	117 242	15 992

a Some observations and variants relate to multiple diseases.

## Supplementary Material

Supplementary Table 2

Supplementary Tutorial
