# Supplementary material for: PGMD: a comprehensive manually curated pharmacogenomic database
Source: Pharmacogenomics J. 2015 May 5;16(2):124–8. doi: 10.1038/tpj.2015.32 (PMC4819767; doi:10.1038/tpj.2015.32)
Supplement: Supplementary Table 1 [file tpj201532x1.doc]

**Supplementary Table 1. Annotation fields of PGMD records as presented in Genome Trax.**

| **Name of the field** | **Description** |
| --- | --- |
| accession | An accession number that is unique for this variant in its pharmacogenomic context  cardinality  1  MySQL type  TEXT  examples  GV000003898  GV (Genome variation) identifiers for single location variants.  HP000000518  HP (Haplotype) identifiers for haplotypes and diplotypes. |
| age | Describes the age of the case group  cardinality  0..1  MySQL type  TEXT  examples  42  32-58  Age can be represented as a range, lower age - upper age of the group  >35  Age can be represented with qualifiers like '<', '>', '<=', '>=', etc |
| amino_acid | Amino acid change, called by snpEff. From snpEff documentation - Amino acid change: old_AA AA_position/new_AA  cardinality  0..*  MySQL type  TEXT  examples  E30K |
| baseline_genotype_ind | In genetic association studies, one allele/genotype/haplotype/diplotype is often considered as a baseline against which the others are compared. For example, the genotype G/G is the baseline against which the genotypes G/C and C/C show "Decreased clearance of metabolite". The baseline is not necessarily the most common case in the population under study, or the one listed in the reference build of the human genome, although this often may be the case. If there was no group for this observation, then the field is empty.  cardinality  0..1  MySQL type  VARCHAR (4)  accepted values  TRUE  The allele/genotype/haplotype/diplotype acts as the baseline in a group of observations.  N/A  The allele/genotype/haplotype/diplotype does not act as the baseline in a group of observations. |
| brief | Genotype:Phenotype. See description of those fields for more detail.  cardinality  0..1  MySQL type  TEXT  examples  C/G or C/C:Decreased risk of drug-induced extrapyramidal symptoms  Short/Short:Decreased risk of drug-induced weight gain |
| cases  (pgmd_cases) | The total number of cases studied for this particular observation.  cardinality  0..1  MySQL type  BIGINT  examples  18 |
| comments  (pgmd_comments) | Free text annotation about sample details, statistical tests, corrections used, etc, that did not fit any of the other categories. The comments are prefixed by a classification tag as follows:   - Primary statistical information - Secondary statistical information - Details about replication - Additional sample details - Variation details - Genotype, haplotype or diplotype details - Additional details - External reference   cardinality  0..*  MySQL type  TEXT  examples  Significance is based on two-tailed Fisher's exact test |
| confidence_interval  (pgmd_confidence_interval) | Confidence interval for the OR (95%) for a particular genotype.  cardinality  0..1  MySQL type  TEXT  examples  1-6.09  0.01-0.66 |
| controls  (pgmd_controls) | The total number of controls studied for this particular observation.  cardinality  0..1  MySQL type  BIGINT  examples  18 |
| disease | Describes the associated disease, if any, in individuals from the case population, by MeSH term (if there is a matching MeSH term).  cardinality  0..*  MySQL type  TEXT  examples  Schizophrenia |
| disease_mesh_id | MeSH-id(s) for the disease term(s) from disease.  cardinality  0..*  MySQL type  TEXT  primary field  disease  examples  D012559  D004827 |
| drug | The name of the compound, drug, substance, drug class or treatment therapy under investigation. Names for actual drugs are taken in order of preference from Drugbank, Pubchem compound, Pubchem substance, and MeSH. The identifiers given in the fields drugbank_id, pubchem_cid, and drug_mesh_id correspond to the names given here, in the same order. This field contains all drugs given to patients, even if the study does not indicate a specific association with each drug. For a list of only the drugs that were under investigation and shown to affect the observed phenotype, see focus_drug. MeSH drug classes are derived from the [therapeutics](http://www.nlm.nih.gov/cgi/mesh/2012/MB_cgi?mode=%26term=Therapeutics%26field=entry) tree of MESH. Drugs, drug classes or treatments that were applied to different study groups are separated by the pipe symbol (|), names within such a group are separated by semicolon (;). This means the field can contain several lists of drugs, drug classes or treatments if there were different patient study groups.  cardinality  1..*  MySQL type  TEXT  examples  Risperidone |
| drug_mesh_id  (pgmd_drug_mesh_id) | The MeSH ID(s) from the Chemicals and Drugs and the Therapeutics sections of MeSH for drug class or drug classes, and treatment or treatments applied. The order is the same as the order of names in drug, with empty positions where no ID is known.  cardinality  0..*  MySQL type  BIGINT  primary field  drug  examples  5073 |
| drugbank_id | The drugbank ID(s) for the compound or compounds applied. The order is the same as the order of names in drug, with empty positions where no ID is known.  cardinality  0..*  MySQL type  TEXT  primary field  drug  examples  DB00333 |
| ensembl_id  (ensembl) | External identifier. Ensembl gene ID for the gene. There may be several identifiers. If there are Locus Reference Genomic available for the gene, in addition to the ENSG identifiers there may be LRG_ identifiers. Also, if there are fix patches for the gene or the gene is variable, there may be additional identifiers.  cardinality  0..*  MySQL type  TEXT |
| entrez_gene_id  (entrez) | External identifier. Entrez gene ID for the gene  cardinality  0..*  MySQL type  BIGINT |
| ethnicity  (pgmd_ethnicity) | Describes the ethnicity of the cases by MeSH term.  cardinality  0..*  MySQL type  TEXT  examples  European Continental Ancestry Group |
| ethnicity_mesh_id  (pgmd_ethnicity_mesh_id) | MeSH-id(s) for the ethnicity term(s) from ethnicity.  cardinality  0..*  MySQL type  TEXT  primary field  ethnicity  examples  D044465 |
| evidence  (pgmd_evidence) | Describes the level of evidence, to aid users as a filter. The classification follows the scheme recommended by PharmGKB.  cardinality  1  MySQL type  VARCHAR (40)  accepted values  Molecular Assay  Pharmacokinetics  Genetic variation in processes involved in the absorption, distribution, metabolism, or elimination of a drug can result in changes in drug availability.  Pharmacodynamics and Drug Response  Genetic variation in drug targets can cause measurable differences in the response of an organism to a drug.  Molecular and Cellular Functional Assays  Genetic variation can alter results of molecular and cellular functional assays, and this may correlate with variations in the organism's drug response.  Clinical Outcome  Genetic variations in the response to drugs can cause measurable differences in clinical endpoints such as rates of cure, morbidity, side effects, and death. |
| focus_disease  (pgmd_focus_disease) | The disease that was the focus of the study. The difference between the focus_disease and disease field is that the disease field contains an exhaustive list of all diseases that the patients may have, regardless of whether or not the drug in question was targeting all of those diseases.  cardinality  0..*  MySQL type  TEXT  examples  HIV Infections, Hepatitis C |
| focus_drug  (pgmd_focus_drug) | All the drugs, drug classes or treatments that were the focus of the study, irrespective of co-medications used. This is a subset of the list in drug, consisting only of those which were shown to affect observed phenotypes.  cardinality  0..*  MySQL type  TEXT  examples  Risperidone |
| genetic_model  (pgmd_genetic_model) | Describes the genetic model used to analyze genotype-phenotype information from association studies.  cardinality  0..1  MySQL type  VARCHAR (21)  accepted values  Allelic model  General genetic model  Dominant model  Recessive model  Co-dominant model  Multiplicative model  Additive model  Haplotype  Combined effect  Not Applicable  Global  Site-specific effect  Trend  Combined genotype |
| genotype  (pgmd_genotype) | The associated allele, genotype, haplotype or diplotype. If specific genotypes are not specified they are given as structured text. Note that if strictly the position itself has been associated with a phenotype, this field may be empty. A haplotype given here may result in multiple records. See also haplotype_id, site_genotype, non_carrier_ind, het_only_ind. Because of the many different ways to describe genotypes, please refer to the examples section for more detail. Square brackets are given just for readability and can be ignored.  cardinality  0..1  MySQL type  TEXT  examples  G  If a single allele was implicated in a drug response, it will be represented as such, in a haploid manner. (variant_type is SNP).  A/C  Genotypes for a single nucleotide variant are represented by separating the allele by slash, always in alphabetical order. (variant_type is SNP).  A/C or C/C  Two or more genotypes that were classified as having the same effect are separated by an or. (variant_type is SNP).  Ins{TTCAC}  Insertion of a given sequence. (variant_type is Indel).  Del{TC}  Deletion of a given sequence. (variant_type is Indel).  Del{T}Ins{GGC}  An overlapping Indel, in which a sequence was deleted, while simultaneously another sequence was inserted. (variant_type is Indel).  het[Del{TC}]  The het tag indicates that the specified variation must occur in a heterozygous manner; the second copy must not necessarily be reference matching but it must not be the specified variation. (can be any variant_type).  non[Del{TC}]  The non tag indicates that anything other than the specified variation should result in a match. (can be any variant_type).  Del{CFTR}  Deletion of the CFTR gene. (variant_type is Gene deletion).  Dup{CFTR}2  Duplication of the CFTR gene. (variant_type is Gene duplication).  Mul{KRAS}7  Seven-fold multiplication of the CFTR gene. (variant_type is Gene multiplication).  3R{CTTCCA}  VNTR with three copies of the sequence CTTCCA. (variant_type is VNTR).  >3R{CTTCCA}  VNTR with more than 3 copies of the sequence CTTCCA. (variant_type is VNTR).  <3R{CTTCCA}  VNTR with less than three copies of the sequence CTTCCA. (variant_type is VNTR).  19R  VNTR with nineteen copies of undefined sequence. (variant_type is VNTR).  3R{CTT}_5G  A VNTR in which a SNP occurred as well. This example is equivalent to 'CTTCGTCTT' where the 5th base is a T>G change. (variant_type is VNTR).  Ref/Ref  Genotype at the position was homozygous reference. (Applies to many variant_types).  G-C-A-T-T  Haplotypes are represented by separating the allele of each variation by a hyphen. (can be any variant_type but will have a non-null haplotype_id).  0 matches (G-C-A-T-T)  Haplotypes in which you must match a certain subset of the sites within that haplotype. In this case, you must not be a the match for any of the given sites. (can be any variant_type but will have a non-null haplotype_id).  >1 match (G-C-A-T-T)  Haplotypes in which you must match a certain subset of the sites within that haplotype. In this case, you must match at least one of the sites. (can be any variant_type but will have a non-null haplotype_id).  2 to 4 matches (G-C-A-T-T)  Haplotypes in which you must match a certain subset of the sites within that haplotype. In this case, you must match at least 2, but no more than 4 sites. (can be any variant_type but will have a non-null haplotype_id).  <5 matches (G-C-A-T-T)  Haplotypes in which you must match a certain subset of the sites within that haplotype. In this case, you must match less than five of the given sites. (can be any variant_type but will have a non-null haplotype_id).  G/G-C/C  Diplotypes are represented by separating the genotype of each variation by a hyphen. This does not necessarily imply phasing. If the diplotypes are phased, | is used instead of /. (can be any variant_type but will have a non-null haplotype_id). |
| genotyping_source  (pgmd_genotyping_source) | Describes the source tissue/cell used for genotyping.  cardinality  0..1  MySQL type  TEXT  examples  Peripheral blood |
| geography  (pgmd_geography) | Describes the geographical provenance of the cases by MeSH term. When the case group includes individuals of more than one geographical region, each term is separated by a semicolon.  cardinality  0..*  MySQL type  TEXT  examples  Spain |
| geography_mesh_id  (pgmd_geography_mesh_id) | Describes the geographical provenance of the cases by MeSH term. When the case group includes individuals of more than one geographical region, each id is separated by a semicolon.  cardinality  0..*  MySQL type  TEXT  primary field  geography  examples  D013030 |
| group_id  (pgmd_group_id) | A number unique within a particular study that is used to group one or more genotypes that were observed for a particular drug response. Each studied variation will belong to one or more observation groups. E.g. Variation G>C leads to three possible genotypes. G/G, G/C, and C/C, studied for a phenotype (e.g. Increased drug toxicity) leading to these three observations being grouped together with a unique group id. If these same 3 genotypes were also studied for another phenotype (e.g. Response rate), those records would be assigned a new group id.  cardinality  1  MySQL type  BIGINT  examples  1  G/G versus G/C versus C/C were compared against each other for drug toxicity impact in one paper, so they are group ID 1.  2  G/G versus G/C versus C/C were also compared against each other for with respect to response rate in the same paper, so these observations will all fall into group 2. |
| haplotype_id  (pgmd_haplotype_id) | If variants co-occuring at multiple sites were shown to lead to a certain phenotype, then those sites would be grouped as a haplotype (we include diplotypes here). A haplotype of the form "A-T-T-G" or a diplotype of the form "A/T-G/C" would be split into individual records per site, and would each share the same haplotype group ID in order to resolve back to the original haplotype.  cardinality  0..1  MySQL type  TEXT  examples  HP000000015-001  The 3 sites of rs2032582, rs1045642, and rs1128503 that that constitute the C-A-C haplotype would each share this ID.  HP000000015-002  The 3 sites of rs2032582, rs1045642, and rs1128503 that that constitute the C-G-T haplotype would each share this ID. |
| hazard_ratio  (pgmd_hazard_ratio) | A measure of how often a particular event happens in one group compared to how often it happens in another group, over time. Often used in clinical trials to measure survival at any point in time in a group of patients who have been given a specific treatment compared to a control group given another treatment treatment or a placebo.  cardinality  0..1  MySQL type  TEXT |
| het_only_ind  (pgmd_het_only_ind) | If this indicator is set to true, it signifies that the value in the Genotype field must be heterozygous in a subject in order for there to be a match. This flag will only be set for records that have been entered at the allele level. (e.g. if Genotype field has a value of "G" and het_only_ind is "TRUE", then a subject who had a "G/T" genotype would be a match, but a subject with a "G/G" genotype would not).  cardinality  0..1  MySQL type  VARCHAR (4)  accepted values  TRUE |
| hgnc | HGNC gene symbol for the gene. If the track describes features that are not directly linked to a gene, and a hgnc symbol is present, it refers to the gene closest downstream or overlapping with the feature.  cardinality  0..*  MySQL type  TEXT |
| hgvs  (pgmd_hgvs) | The Human Genome Variation Society (HGVS) description of the variation, or if not available, free text, following HGVS rules. Sometimes chromosomal coordinates must be specified instead. For more on HGVS nomenclature, see<http://www.hgvs.org/mutnomen/>. An observation can include more than one variation (e.g. for haplotypes and diplotypes).  cardinality  0..*  MySQL type  TEXT  examples  NT_010783.15:g.10634882T>C  The default contig is NT  genedeletion{BRCA1}  CNVs/SVs are described as accurately as possible  chr10:96826971delT  Absolute coordinate representation |
| hyperlink | Link to a report or web-page with more detailed information.  cardinality  0..1  MySQL type  TEXT |
| max_haplotype_matches  (pgmd_max_haplotype_matches) | When haplotype_id is populated (the variation is a haplotype), this field may be populated. A populated value means that of the sites in a haplotype, y number of these sites must be a positive match to be a match for the given observation. See also min_haplotype_matches.  cardinality  0..1  MySQL type  BIGINT  examples  3  In a haplotype of 5 sites (e.g. G-T-C-A-T), if a subject matches 3 of those sites (e.g. G-T-C-G-G) then they will be a match for this observation, but if they match 4 or all of those sites, they will not. |
| metabolizer  (pgmd_metabolizer) | Metabolizer status of the haplotype or diplotype.  cardinality  0..1  MySQL type  VARCHAR (25)  accepted values  Slow Metabolizers  Poor Metabolizers  Intermediate Metabolizers  Moderate Metabolizers  Rapid Metabolizers  Extensive Metabolizers  Ultra-rapid Metabolizers  Normal Metabolizers |
| min_haplotype_matches  (pgmd_min_haplotype_matches) | When haplotype_id is populated (the variation is a haplotype), this field may be populated. A populated value means that of the sites in a haplotype, x number of these sites must be a positive match to be a match for the given observation. See also max_haplotype_matches.  cardinality  0..1  MySQL type  BIGINT  examples  0  A value of 0 means that if a subject has no matches for the given haplotype, they are a match for the given observation.  2  In a haplotype of 3 sites (e.g. A-T-T), if a subject has a match for only 1 or 0 of those sites, then they will not be a match here. |
| named_variation  (pgmd_named_variation) | Regularly known as "Star alleles". In named variants, genotypes are represented with gene names followed by allele designation e.g. CYP2C9*1/CYP2C9*1. We have resolved these star alleles to the actual site(s) that make them up. In cases where a publication has specified the sites that they considered for a given named variation, we use just those sites. In cases where a publication has simply referred to a variation by its star allele nomenclature, we have resolved that variation to the sites for that named variation that differ from the wild-type (*1) allele. In cases that were reported as heterozygous (e.g. CYP2C9*1/*3), rather than considering all sites that have been observed for *1, we only consider the sites where *3 has varied. For homozygous reference, (*1/*1), we consider all sites that have seen variation within the set of named variations. To see what the genotypes were resolved to, see also genotype.  cardinality  0..1  MySQL type  TEXT  examples  CYP2C9*1/CYP2C9*6 |
| nearby_genes  (pgmd_nearby_genes) | In addition to the single HGNC gene symbol that gets assigned in the HGNC field, this contains a more complete description of gene symbols that are associated with the variant, based on the variant's position in the genome. Contains a single HGNC symbol, if the variant overlaps a single gene. When a variant overlaps multiple genes, each gene symbol is separated by a semicolon (and the strand of the gene may be given as (+) or(-). If the site is intergenic, it will contain the 4 nearest genes (5' and 3' on the positive and negative strands, and a signed distance from each gene, - meaning towards smaller genomic coordinates, + towards larger ones, separated by comma.  cardinality  0..1  MySQL type  TEXT  examples  SLCO1B3  A single gene overlaps the variant.  SLCO1B3 (+), SLCO1B4 (-)  Two genes, on opposite strands overlap the variant.  SLCO1B3 (+) -17000, SLCO1B4 (+) +42000, SLCO1B5 (-) -10000, SLCO1B6 (-) +27000  The variant is intergenic, the four neighboring genes are given with strand and distance. |
| non_carrier_ind  (pgmd_non_carrier_ind) | If this indicator is set to true, it signifies that the respective record applies only to subjects that do not carry the allele, genotype, haplotype, or diplotype specified in the genotype field. (e.g. if Genotype field has a value of "A" and non_carrier is "true", then a subject who had a "T/T" genotype would be a match, but a subject with a "T/A" genotype would not). See also genotype.  cardinality  0..1  MySQL type  VARCHAR (4)  accepted values  TRUE  N/A |
| obsid  (pgmd_obsid) | A globally unique identifier for each observation that has been curated. This is assigned based on the site/allele/genotype/haplotype/diplotype that has been associated with a specific effect. An observation may span number of genomic sites depending on the number of variants acting together as a haplotype.  cardinality  1..*  MySQL type  BIGINT  examples  3361673 |
| odds_ratio  (pgmd_odds_ratio) | The odds that an individual with this genetic profile will actually exhibit this phenotype.  cardinality  0..1  MySQL type  TEXT  examples  0.29  2.47 |
| p_value  (pgmd_p_value) | P-value for a particular genotype as given in the reference.  cardinality  0..1  MySQL type  TEXT  examples  0.05  <0.01  If the reference states a p-value as being lower than a given value, then the value is prefixed by the 'less than' sign. |
| phenotype  (pgmd_phenotype) | A qualitative description of the impact of genetic variation on drug response.  cardinality  1  MySQL type  TEXT  examples  Decreased risk of drug-induced extrapyramidal symptoms. |
| phenotype_category  (pgmd_phenotype_category) | The general category of drug response, chosen from our controlled vocabulary of phenotypes.  cardinality  0..1  MySQL type  TEXT  examples  extrapyramidal symptoms |
| phenotype_detail  (pgmd_phenotype_detail) | A quantitative description of the impact of genetic variation on drug response, typically detailing the fraction of subjects with the specified genetic profile that exhibited the given response, and further detail on that response that would be given in the Phenotype field.  cardinality  0..1  MySQL type  TEXT  examples  Oral clearance of Verapamil for G/G-C/C diplotype is 452.2 +/- 188.6l/hr |
| pmid | Pubmed ID of the reference from which the information was taken.  cardinality  0..*  MySQL type  BIGINT |
| pubchem_cid | The PubChem compound CID(s) for the compound or compounds administered. The order is the same as the order of names in drug, with empty positions where no ID is known.  cardinality  0..*  MySQL type  BIGINT  primary field  drug  examples  5073 |
| ref_id | The reference from where this curation came. *See also ref_type.  cardinality  1  MySQL type  BIGINT  primary field  ref_type  examples  11586955  A PubMed Identifier  Abacavir-03/04/14  The abacavir FDA drug label, curated March 4, 2014 |
| ref_type | The type of reference that ref_id refers to.  cardinality  1..*  MySQL type  VARCHAR (9)  accepted values  pubmedid  Source is from PubMed (http://www.ncbi.nlm.nih.gov/pubmed/).  fda_label  Source is an FDA drug label (http://www.accessdata.fda.gov/scripts/cder/drugsatfda/). |
| reference_allele  (pgmd_reference_allele) | The allele found in the corresponding human reference assembly.  cardinality  0..1  MySQL type  TEXT  examples  T |
| registry_identifiers  (pgmd_registry_identifiers) | Official approval or recommendation like a clinicaltrials.gov number. Currently included are ClinnicalTrials.gov, EudraCT, NCCTG, EDCTP, ACTG, ACTR, Chinese Clinical Trial Registry Number, ISRCTN Register, UMIN-CTR registration, and Netherlands trial registry.  cardinality  0..*  MySQL type  TEXT  examples  ClinicalTrials.gov Identifier:NCT00006206  ACTR Number:12610000270011 |
| relative_risk  (pgmd_relative_risk) | The likelihood that an individual with this genetic profile will exhibit this phenotype versus the likelihood that someone who does not have this genetic profile will exhibit this phenotype.  cardinality  0..1  MySQL type  TEXT |
| rsid | dbSNP ID number, if available.  cardinality  0..*  MySQL type  TEXT  examples  rs6280 |
| sample_size  (pgmd_sample_size) | Describes the total sample size in the study. Sum of cases and controls across all genotypes.  cardinality  0..1  MySQL type  BIGINT  examples  111 |
| sex  (pgmd_sex) | Describes the gender of the individuals in the study.  cardinality  1  MySQL type  VARCHAR (11)  accepted values  Female  Male  Mixed  Unspecified |
| site_genotype  (pgmd_site_genotype) | The allele or genotype for a single position that has been derived from the genotype column. In the case of haplotypes, genotype will represent the full haplotype for an observation (e.g. T-A-G), which will then be split into multiple records in site_genotype, linked through the obsid and haplotype group id to resolve the overall haplotype. In the case of multiple genotypes that were all associated with the same drug response, genotype will represent the grouped genotypes (e.g. "A/A or A/G"), which will then be split into multiple records, but will share the same observation id (obsid).  cardinality  0..1  MySQL type  TEXT  examples  T  If Genotype is simply 'T', site_genotype will be the same. If Genotype is 'T or C', this observation will be split into 2 records, having site_genotype of 'T' for entry 1, and 'C' for 2. If Genotype is 'T-G-A' (a haplotype of 3 sites), this observation will be split into 3 records, having site_genotype of 'T' for the first site, 'G' for the second, and 'A' for the third.  A/C  If Genotype is simply 'A/C', site_genotype will be the same. If Genotype is 'A/C or C/C', this observation will be split into 2 records, having site_genotype of 'A/C' for entry 1, and 'C/C' for 2. If Genotype is 'A/C-G/G-A/A' (a diplotype of 3 sites), this observation will be split into 3 records, having site_genotype of 'A/C' for the first site, 'G/G' for the second, and 'A/A' for the third.  >16R{CA}/>16{CA}  VNTRs are maintained as-is due to the complexity of expanding VNTRs in which a range of repeats was specified.  16R{CA}  VNTRs are maintained as-is due to the complexity of expanding VNTRs in which a range of repeats was specified.  <16R{CA}  VNTRs are maintained as-is due to the complexity of expanding VNTRs in which a range of repeats was specified.  ->TTCAC  A Genotype of 'Ins{TTCAC}' (insertion of TTCAC sequence) will be represented as such.  TC>-  A Genotype of 'Del{TC}' (deletion of TC sequence) will be represented as such.  T>GGC  A Genotype of 'Del{T}Ins{GGC}' (overlapping deletion of T and insertion of GGC at the same site) will be represented as such. |
| study_design  (pgmd_study_design) | Describes the study type. In the case of several possible terms, the most specific one that indicates the highest predictive power will be used. For example, for randomized, controlled clinical trials, if the reference mentions several stages, then the highest stage will be used.  cardinality  1  MySQL type  TEXT  examples  Clinical Trial (general, phases unknown) |
| treatment_detail  (pgmd_treatment_detail) | Dose, duration and the route of administration of the compounds used for treatment in the case group.  cardinality  0..1  MySQL type  TEXT  examples  Methotrexate was given to all patients for at least 3 months, with an initial dose of 4-5 mg/2 months/week and then up-to maximal dosage of 10 mg/2 months/week by oral mode. Prednisolone was also given to 89 patients and nine patients were supplemented with Folic acid |
| uniprot_acc  (uniprot) | External identifier. UniProt accession number for the protein. If there are several possible sequences, the canonical sequence and accession number is used.  cardinality  0..*  MySQL type  TEXT |
| variant_class  (pgmd_variant_class) | Class of variant, as predicted by snpEff, based on the "canonical" transcript. Values include missense, nonsense, synonymous, and frameshift; please refer to snpEff for [a full vocabulary](http://snpeff.sourceforge.net/SnpEff_manual.html#eff).  cardinality  0..*  MySQL type  TEXT |
| variant_type  (pgmd_variant_type) | Variant type.  cardinality  1..*  MySQL type  VARCHAR (19)  accepted values  SNP  Single Nucleotide Polymorphism  Indel  Insertion, Deletion, or Overlapping Insertion and Deletion  VNTR  Variable Number of Tangent Repeats  Gene deletion  Deletion of entire gene  Gene duplication  Duplication of gene  Gene amplification  Amplification event  Gene multiplication  Multiplication event  Polymorphism  Applies to polymorphisms of unknown type. |
