# Supplementary material for: PGMD: a comprehensive manually curated pharmacogenomic database
Source: Pharmacogenomics J. 2015 May 5;16(2):124–8. doi: 10.1038/tpj.2015.32 (PMC4819767; doi:10.1038/tpj.2015.32)
Supplement: Supplementary Table 2 [file tpj201532x2.doc]

**Supplementary Table 2.** Example of PGMD data download in tsv format.

| **Field** | **Allele** | | |
| --- | --- | --- | --- |
| chr | chr22 | chr22 | chr22 |
| start | 37632770 | 37632770 | 37632770 |
| end | 37632770 | 37632770 | 37632770 |
| hgnc | RAC2 | RAC2 | RAC2 |
| rsid | rs13058338 | rs13058338 | rs13058338 |
| pgmd_site_genotype | T/T | A/A | A/T |
| pgmd_genotype | T/T | T/A or A/A | T/A or A/A |
| pgmd_non_carrier_ind | N/A | N/A | N/A |
| pgmd_het_only_ind | N/A | N/A | N/A |
| strand | + | + | + |
| pgmd_reference_allele | T | T | T |
| pgmd_phenotype | Typical risk of acute cardiotoxicity | Increased risk of acute cardiotoxicity | Increased risk of acute cardiotoxicity |
| pgmd_phenotype_category | adverse cardiovascular events | adverse cardiovascular events | adverse cardiovascular events |
| pgmd_phenotype_detail | Out of 250 subjects with corresponding genotypes, 15 subjects developed acute cardiotoxicity | Out of 198 subjects with T/A or A/A genotype, 23 subjects with T/A and 5 subjects with A/A genotype developed acute cardiotoxicity | Out of 198 subjects with T/A or A/A genotype, 23 subjects with T/A and 5 subjects with A/A genotype developed acute cardiotoxicity |
| pgmd_focus_disease | Lymphoma, Non-Hodgkin | Lymphoma, Non-Hodgkin | Lymphoma, Non-Hodgkin |
| pgmd_focus_drug | Doxorubicin | Doxorubicin | Doxorubicin |
| pgmd_group_id | 454 | 454 | 454 |
| pgmd_obsid | 3408385 | 3408386 | 3408386 |
| pgmd_haplotype_id | N/A | N/A | N/A |
| pgmd_sample_size | 448 | 448 | 448 |
| pgmd_cases | 250 | 198 | 198 |
| pgmd_controls | N/A | N/A | N/A |
| pgmd_p_value | N/A | 0.025 | 0.025 |
| pgmd_odds_ratio | N/A | 1.7 | 1.7 |
| pgmd_confidence_interval | N/A | N/A | N/A |
| pgmd_relative_risk | N/A | N/A | N/A |
| pgmd_hazard_ratio | N/A | N/A | N/A |
| pgmd_baseline_genotype_ind | TRUE | N/A | N/A |
| pgmd_comments | Primary statistical information: Significance is based on multiple logistic regression analysis adjusted for age, gender, cumulative dose administered until development of ATC, and dosing interval; Secondary statistical information: The association is also significant by Fisher's exact test, with p=0.005 and also by Freidlin test, with p=0.002. | Primary statistical information: Significance is based on multiple logistic regression analysis adjusted for age, gender, cumulative dose administered until development of ATC, and dosing interval; Secondary statistical information: The association is also significant by Fisher's exact test, with p=0.005 and also by Freidlin test, with p=0.002. | Primary statistical information: Significance is based on multiple logistic regression analysis adjusted for age, gender, cumulative dose administered until development of ATC, and dosing interval; Secondary statistical information: The association is also significant by Fisher's exact test, with p=0.005 and also by Freidlin test, with p=0.002. |
| pgmd_evidence | Pharmacodynamics and Drug Response | Pharmacodynamics and Drug Response | Pharmacodynamics and Drug Response |
| pgmd_genetic_model | Dominant model | Dominant model | Dominant model |
| ref_id | 16330681 | 16330681 | 16330681 |
| ref_type | pubmedid | pubmedid | pubmedid |
| pgmd_study_design | Randomized Controlled Clinical Trial (Clinical Trial, phase III) | Randomized Controlled Clinical Trial (Clinical Trial, phase III) | Randomized Controlled Clinical Trial (Clinical Trial, phase III) |
| pgmd_genotyping_source | Peripheral lymphocytes | Peripheral lymphocytes | Peripheral lymphocytes |
| pgmd_metabolizer | N/A | N/A | N/A |
| pgmd_named_variation | N/A | N/A | N/A |
| pgmd_near_gene | N/A | N/A | N/A |
| disease | Lymphoma, Non-Hodgkin | Lymphoma, Non-Hodgkin | Lymphoma, Non-Hodgkin |
| drug | Cyclophosphamide, Prednisone, Vincristine, Doxorubicin, Etoposide, rhG-csf | Cyclophosphamide, Prednisone, Vincristine, Doxorubicin, Etoposide, rhG-csf | Cyclophosphamide, Prednisone, Vincristine, Doxorubicin, Etoposide, rhG-csf |
| pgmd_variant_class | INTRON | INTRON | INTRON |
| amino_acid | N/A | N/A | N/A |
| pgmd_variant_type | SNP | SNP | SNP |
| pgmd_ethnicity | European Continental Ancestry Group | European Continental Ancestry Group | European Continental Ancestry Group |
| pgmd_geography | Germany | Germany | Germany |
| pgmd_age | N/A | N/A | N/A |
| pgmd_sex | Mixed | Mixed | Mixed |
| pgmd_treatment_detail | Sample consists of patients from a prospective multicenter randomized phase III trial where they are randomly assigned to receive either standard CHOP-21 regimen (which includes i. v. administration of 750 mg/m(2) of cyclophosphamide, 50 mg/m(2) of doxorubicin and 2 mg of vincristine on day1 and 100 mg/d per os of prednisone on days 1 to 5), CHOEP-21 (addition of 100 g/m(2) of etoposide on day 1 to 3 with standard CHOP-21 regimen), shortening to 2-week intervals with the use of recombinant human granulocyte colony-stimulating factor (rhGCSF, CHOP-14), or both (CHOEP-14). Patients with anthracycline-induced cardiotoxicity (ACT) presented as arrhythmias (acute ACT) or congestive heart failure (chronic ACT). | Sample consists of patients from a prospective multicenter randomized phase III trial where they are randomly assigned to receive either standard CHOP-21 regimen (which includes i. v. administration of 750 mg/m(2) of cyclophosphamide, 50 mg/m(2) of doxorubicin and 2 mg of vincristine on day1 and 100 mg/d per os of prednisone on days 1 to 5), CHOEP-21 (addition of 100 g/m(2) of etoposide on day 1 to 3 with standard CHOP-21 regimen), shortening to 2-week intervals with the use of recombinant human granulocyte colony-stimulating factor (rhGCSF, CHOP-14), or both (CHOEP-14). Patients with anthracycline-induced cardiotoxicity (ACT) presented as arrhythmias (acute ACT) or congestive heart failure (chronic ACT). | Sample consists of patients from a prospective multicenter randomized phase III trial where they are randomly assigned to receive either standard CHOP-21 regimen (which includes i. v. administration of 750 mg/m(2) of cyclophosphamide, 50 mg/m(2) of doxorubicin and 2 mg of vincristine on day1 and 100 mg/d per os of prednisone on days 1 to 5), CHOEP-21 (addition of 100 g/m(2) of etoposide on day 1 to 3 with standard CHOP-21 regimen), shortening to 2-week intervals with the use of recombinant human granulocyte colony-stimulating factor (rhGCSF, CHOP-14), or both (CHOEP-14). Patients with anthracycline-induced cardiotoxicity (ACT) presented as arrhythmias (acute ACT) or congestive heart failure (chronic ACT). |
| pgmd_hgvs | NT_011520.12:g.17023339T>A | NT_011520.12:g.17023339T>A | NT_011520.12:g.17023339T>A |
| feature | T/T:Typical risk of acute cardiotoxicity | T/A or A/A:Increased risk of acute cardiotoxicity | T/A or A/A:Increased risk of acute cardiotoxicity |
| hyperlink | https://portal.biobase-international.com/cgi-bin/knowledgebase/idb/1.0/get.cgi?GV000000010 | https://portal.biobase-international.com/cgi-bin/knowledgebase/idb/1.0/get.cgi?GV000000010 | https://portal.biobase-international.com/cgi-bin/knowledgebase/idb/1.0/get.cgi?GV000000010 |
| accession | GV000000010 | GV000000010 | GV000000010 |
| ID | 1514118 | 1514129 | 1514142 |
| drugbank_id | DB00531,DB00635,DB00541,DB00997,DB00773,N/A | DB00531,DB00635,DB00541,DB00997,DB00773,N/A | DB00531,DB00635,DB00541,DB00997,DB00773,N/A |
| pubchem_cid | 2907,5865,5978,31703,36462,N/A | 2907,5865,5978,31703,36462,N/A | 2907,5865,5978,31703,36462,N/A |
| uniprot | P15153 | P15153 | P15153 |
| entrez | 5880 | 5880 | 5880 |
| ensembl | ENSG00000128340 | ENSG00000128340 | ENSG00000128340 |
| disease_mesh_id | D008228 | D008228 | D008228 |
| pgmd_drug_mesh_id | N/A | N/A | N/A |
| pgmd_ethnicity_mesh_id | D044465 | D044465 | D044465 |
| pgmd_geography_mesh_id | D005858 | D005858 | D005858 |
| pgmd_registry_identifiers | N/A | N/A | N/A |
