# Supplementary material for: PGMD: a comprehensive manually curated pharmacogenomic database
Source: Pharmacogenomics J. 2015 May 5;16(2):124–8. doi: 10.1038/tpj.2015.32 (PMC4819767; doi:10.1038/tpj.2015.32)
Supplement: Supplementary Tutorial [file tpj201532x3.pdf]

Supplementary tutorial to:

PGMD: a comprehensive manually curated pharmacogenomic database

Alexander Kaplun, Jennifer D. Hogan, Frank Schacherer, Arul P. Peter, Sudheer Krishna, Burkhard R. Braun, Rekha Nambudiry, Nitu MG, Rohan Mallelwar, Adem Albayrak

BIOBASE GmbH, Halchtersche Strasse 33, 38304 Wolfenbüttel, Germany

PGMD™ Online Interface search options

4 ways to search

- By variant
- By drug
- By disease
- By gene

Search by variant

Searching by variant is the most direct approach when you are interested in a specific variant and know the dbSNP accession (rsID), the resulting amino acid change, or the genomic coordinates for the variant. For example, you've read a recent publication describing a NOD2 variant (rs2066844) as a marker for early intensive treatment of Crohn's disease. To quickly find the report for this variant, select the Variants radio button in the search options box, select the Identifier radio button, enter the accession in the search field and click the search button. The matched variant, and any haplotypes containing the matched variant, will be listed in the search results. A short overview is provided including the gene(s) that the variant falls within or is surrounded by, as well as the drug that is affected by the variant and the disease context of the variant-drug association. Click the hyperlinked variant name to navigate to the variant report which provides details of the phenotype observed for the variant. If you have a list of accession to search by, use the Upload a list of variants option.

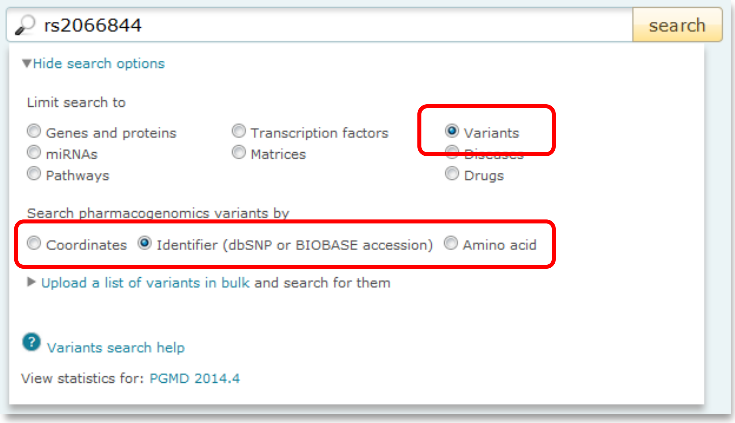

Know the amino acid change caused by the variation? Select the Amino acid change radio button and enter the amino acid change (three letter format, i.e. Arg702Trp) in the search field.

Want to find the variant at a specific genomic coordinate or the list of variants within a genomic coordinate range? Select the Coordinate radio button and enter your coordinates in .bed format in the search field. If you have a list of coordinates to search by, use the Upload a list of variants option.

To quickly obtain a list of all genes, drugs or diseases associated with the variant use the search within results option within the results header. The resulting list of variants can be downloaded in txt format using Export these results link.

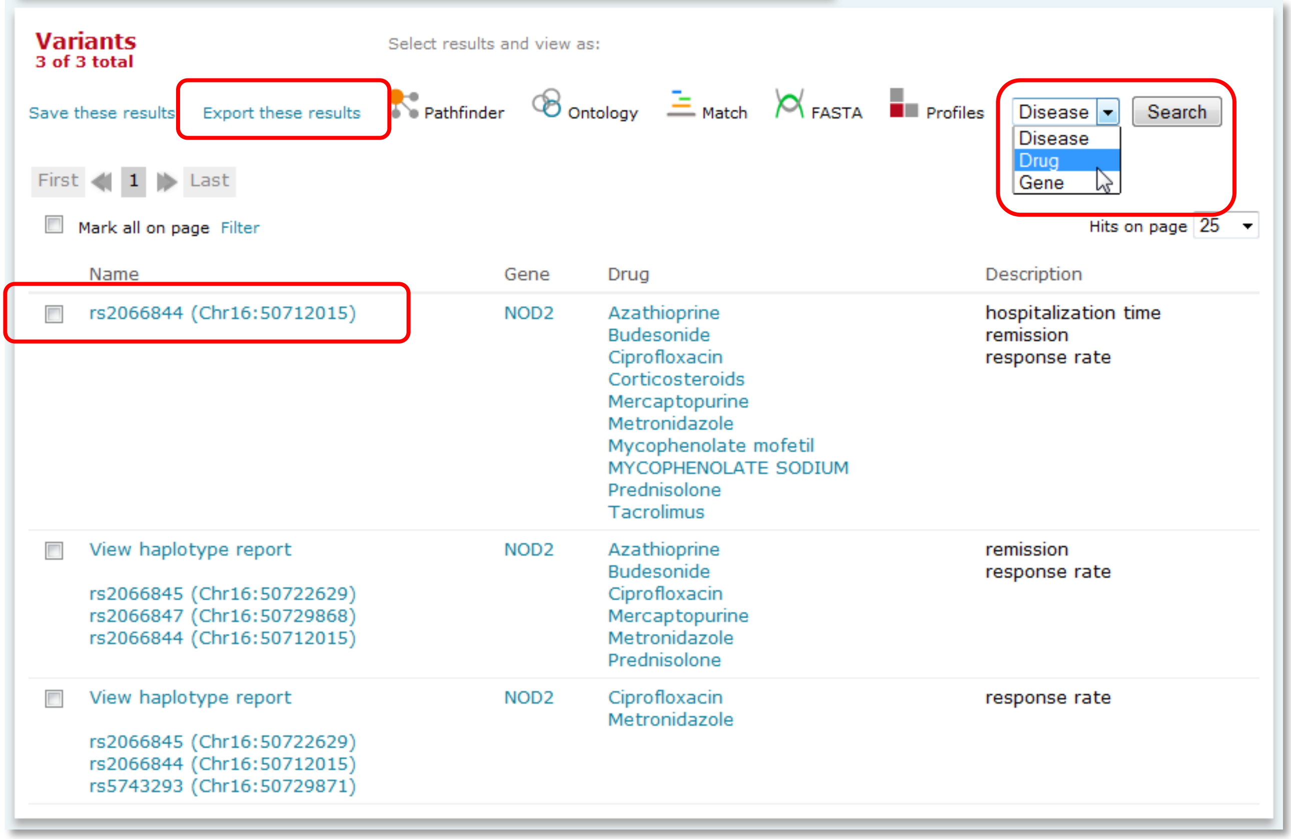

Search by drug

Searching by drug is most appropriate when you are interested in learning about the drug itself (including information about the protein(s) it target(s), which enzyme(s) metabolize the drug, as well as general properties) or are interested in identifying all or a subset of variants that have been shown to affect response to the drug. To quickly find the report for a drug, select the Drugs radio button in the search options box, enter the drug name in the search field and click the search button. The matched drug will be listed in the search results. A short description of the drug (provided by DrugBank) is provided when available. Click the hyperlinked drug name to navigate to the drug report which provides details of the drug, its targets, its metabolizers and the variants that influence patient response.

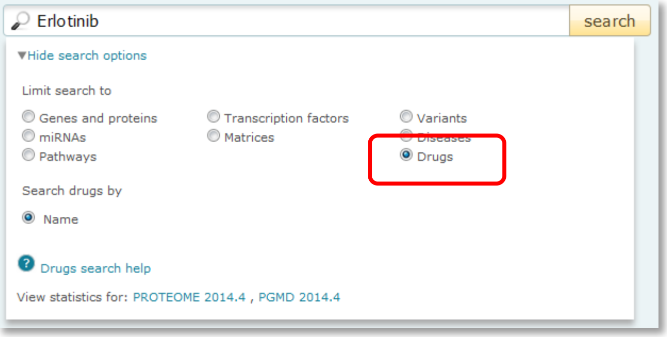

To quickly obtain a list of all variants associated with the drug use the search within results option within the results header. The resulting list of variants can be downloaded in txt format using Export these results link.

Search by disease

Searching by disease is most appropriate when you are interested in learning about the disease itself (including information about the genes and miRNAs associated with the disease as well as clinical trials underway or already completed) or are interested in identifying all or a subset of variants that have been shown to affect response to a drug administered in the context of the disease. To quickly find the report for a disease, select the Diseases radio button in the search options box, enter the disease name in the search field and click the search button. The matched disease will be listed in the search results. A short description of the disease (provided by MeSH) is provided when available. Click the hyperlinked disease name to navigate to the disease report which provides details of the disease, the genes and miRNAs involved, ongoing and completed clinical trials and the variants associated with altered drug response in the treatment of the disease.

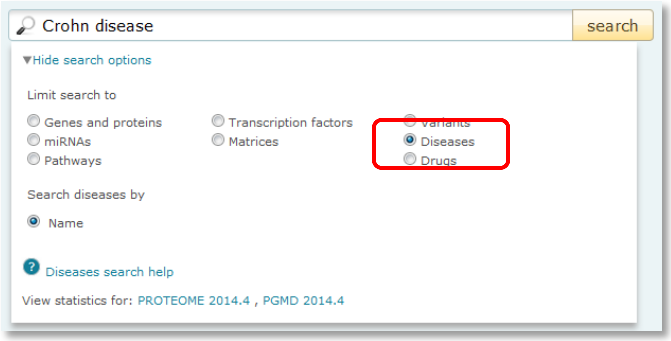

To quickly obtain a list of all variants associated with drug response in the treatment of the disease use the search within results option within the results header. The resulting list of variants can be downloaded in txt format using Export these results link.

Search by gene

Searching by gene is most appropriate when you are interested in learning about a particular gene or are interested in identifying all or a subset of pharmacogenomic variants that fall within the gene. To quickly find the report for a gene, select the Genes and proteins radio button in the search options box, select the Name radio button, enter the gene name in the search field and click the search button. The matched gene will be listed in the search results. A short description of the gene (curated by BIOBASE curators) is provided. Click the hyperlinked gene name to navigate to the locus report which provides details about the gene and its encoded protein(s) and the pharmacogenomic variants that fall within the gene.

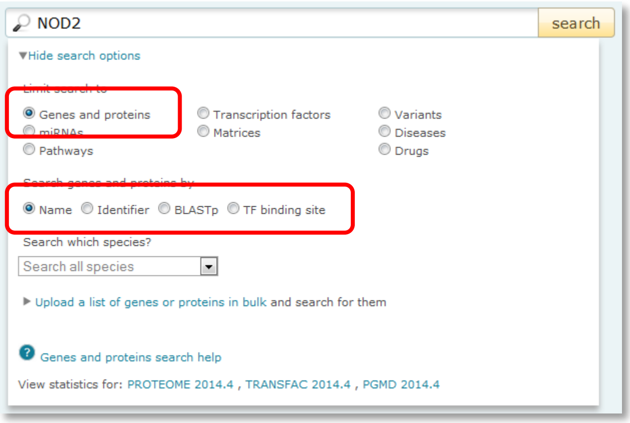

To quickly obtain a list of all variants that fall within a gene use the search within results option within the results header. The resulting list of variants can be downloaded in txt format using Export these results link.

Variant report

BIOBASE BIOLOGICAL DATABASES

Variant Report

rs2066844 (Chr16:50712015)

logout help

Table of Contents

Introduction what is this?

Variant Overview

1

Gene : NOD2

Variant type : SNP

Variant class : missense, upstream_gene

Amino acid change : Arg702Trp

Position : Chr16:50712015

Reference allele : C

Allele frequency(C) : calculated_all 0.970395

Allele frequency(T) : calculated_all 0.030729

HGVS description :
NT_010498.15:g.4360125C>T,NM_001293557.1:c.2023C>T,NM_022162.2:c.2104C>T,NP_001280486.1:p.Arg675Trp,NP_071445.1:p.Arg702Trp

Corticosteroids, Mycophenolate mofetil, MYCOPHENOLATE SODIUM, Tacrolimus studies

Study 1

2

Population : European Continental Ancestry Group

Age : (Study group(s): 1) Mean 49.7

Sex : Mixed

Study design : Clinical Trial (general, phases unknown)

Genetic model : Dominant model

Source used for genotyping : Blood

Total sample size : 75

PMID : 23175667

Treatment : Sample consists of de novo adult renal transplant recipients who received regimen consisted of tacrolimus, corticosteroids, and mycophenolate mofetil or sodium. Oral treatment was started within 24 hours after surgery unless there were complications. All the patients received tacrolimus orally as the primary immunosuppression drug at an initial dose of 0.1-0.2 mg/kg/day divided into two doses. The dose of tacrolimus was individualized by therapeutic drug monitoring to maintain the target blood trough concentration of 10-15 ng/ml.

Show 5 entries Search: 3 Show / hide columns

Genotype	Study group ID	Phenotype	Phenotype details	Named variant	Cases	p-val
C/T or T/T	1	Typical Hospitalization time	Median hospitalization time in subjects with corresponding genotypes is 9.5 days		71	
C/C	1	Increased hospitalization time	Median hospitalization time in subjects with corresponding genotypes is 13.5 days		4	0.013

Showing 1 to 2 of 2 entries First Previous 1 Next Last

Linkage Disequilibrium what is this?

4

Show 5 entries Search:

Site	Population	D'	r^2
rs10459815	African ancestry in Southwest USA	1	0.002
rs10459815	Chinese in Metropolitan Denver, Colorado	1	0.04
rs10459815	Luhya in Webuye, Kenya	1	0.002
rs10459815	Mexican ancestry in Los Angeles, California	1	0.002
rs10459815	Maasai in Kinyawa, Kenya	1	0.001

Showing 1 to 5 of 1,277 entries First Previous 1 2 3 4 5 Next Last

References (3)

5

Show 5 entries Search:

Sl.No	PMID	Citation
1	23175667	Boso, V., Herrero, M. J., Bea, S., Galiana, M., Marrero, P., Marques, M. R., Hernandez, J., Sanchez-Plumed, J., Poveda, J. L., Alino, S. F., Increased hospital stay and allograft dysfunction in renal transplant recipients with Cyp2c19 AA variant in SNP rs4244285. Drug Metab Dispos 41 (2) 480-7 (2013). Show abstract

Each variant report provides detailed information about a site that has been known to affect drug response in humans.

1 The introduction provides basic information gene, type of variant, snpEff predicted effect, coordinate, reference allele, and an average frequency based off of Exome Variant Server, 1000 Genomes, and HapMap.

2 Studies are grouped by the drug that was administered to patients in a clinical trial, and display how one genotype versus another has been shown to respond to a treatment.

3 A default set of columns is provided in the study tables, but additional information may be selected for display by clicking the “Show/hide columns” option. Link outs to gene, drug, and disease reports are also included.

4 Linkage disequilibrium data, when available from HapMap, is provided organized by population. To limit the data by population type the desired population into the Search box.

5 All references, linked to PubMed or FDA label, are listed in the last table.
